# TASK-2: a K_2P_ K^+^ channel with complex regulation and diverse physiological functions

**DOI:** 10.3389/fphys.2013.00198

**Published:** 2013-07-29

**Authors:** L. Pablo Cid, Hugo A. Roa-Rojas, María I. Niemeyer, Wendy González, Masatake Araki, Kimi Araki, Francisco V. Sepúlveda

**Affiliations:** ^1^Centro de Estudios CientíficosValdivia, Chile; ^2^Universidad Austral de ChileValdivia, Chile; ^3^Centro de Bioinformática y Simulación Molecular, Universidad de TalcaTalca, Chile; ^4^Institute of Resource Development and Analysis, Kumamoto UniversityKumamoto, Japan

**Keywords:** K_2P_ channels, TASK-2 channel, bicarbonate reabsorption, central chemoception, cell volume regulation, chondrocytes

## Abstract

TASK-2 (K_2P_5.1) is a two-pore domain K^+^ channel belonging to the TALK subgroup of the K_2P_ family of proteins. TASK-2 has been shown to be activated by extra- and intracellular alkalinization. Extra- and intracellular pH-sensors reside at arginine 224 and lysine 245 and might affect separate selectivity filter and inner gates respectively. TASK-2 is modulated by changes in cell volume and a regulation by direct G-protein interaction has also been proposed. Activation by extracellular alkalinization has been associated with a role of TASK-2 in kidney proximal tubule bicarbonate reabsorption, whilst intracellular pH-sensitivity might be the mechanism for its participation in central chemosensitive neurons. In addition to these functions TASK-2 has been proposed to play a part in apoptotic volume decrease in kidney cells and in volume regulation of glial cells and T-lymphocytes. TASK-2 is present in chondrocytes of hyaline cartilage, where it is proposed to play a central role in stabilizing the membrane potential. Additional sites of expression are dorsal root ganglion neurons, endocrine and exocrine pancreas and intestinal smooth muscle cells. TASK-2 has been associated with the regulation of proliferation of breast cancer cells and could become target for breast cancer therapeutics. Further work in native tissues and cells together with genetic modification will no doubt reveal the details of TASK-2 functions that we are only starting to suspect.

Members of the K_2P_ family of K^+^ channels underlie the leak conductance that is central in determining the resting membrane potential of all cells. These K^+^ channels have an unusual topology, first noticed in the eight trans-membrane domain (TM) yeast channel subunit TOK, which contains two, rather than a single, pore domains (P). Shortly after TWIK-1, the first mammalian K_2P_ channel was discovered (Lesage et al., 1996). The channel fulfilled the main characteristic of the long known background K^+^ conductance, i.e., that of being open at rest and therefore of driving the membrane potential toward the equilibrium potential for K^+^. The name of the channel was designed to describe functional and structural features in one ingenious acronym: Tandem of P domains in a Weak Inwardly rectifying K^+^ channel. Cloning of TWIK-1 was followed by a flurry of identification/characterization of K_2P_ channels that promptly brought the family to its present strength of 15 mammalian members (Enyedi and Czirják, 2010) and a highly confusing but enduring nomenclature (see Figure 1).

**Figure 1:**
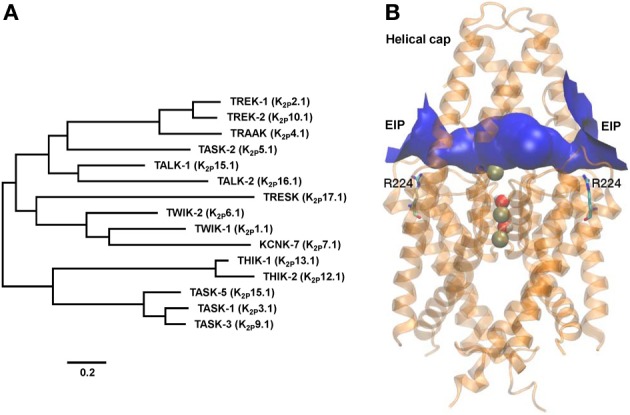
**TASK-2 position within the K_2P_ phylogeny and structural molecular model. (A)** Phylogenetic tree constructed using human K_2P_ channel sequences with the MEGA5 software (www.megasoftware.net) using the maximum likelihood method. The scale bar indicates an evolutionary distance of 0.2 amino acid substitutions per position. Common and International Union of Pharmacology (Goldstein et al., 2005) names are given. **(B)** Modeling the extracellular ion pathway (EIP) of TASK-2 channel and the position of the R224 pH_o_ sensors. A molecular model for the TASK-2 pore based on the structure of TRAAK (PDB ID 3UM7) is shown with both sensing R224 residues facing the EIP. Shown is a ribbon representation with K^+^ ions and H_2_O molecules in the selectivity filter (SF). R224 pH_o_-sensing residues are shown in stick representation. The EIP is drawn as a solid tunnel connecting the extracellular space and the entrance of the SF. HOLE color code is used: blue, radius >1.15 Å. The illustration is taken at the end of a 10-ns MD run.

TASK-2, the K_2P_ channel object of this review, was discovered in 1998 (Reyes et al., 1998). The channel's name was created as an acronym of TWIK-related Acid-Sensitive K^+^ channel 2, reflecting its functional relation through shared extracellular pH (pH_o_) sensitivity to previously discovered TASK-1 (Duprat et al., 1997). A similar reason brought the later described TASK-3 into this group (Kim et al., 2000; Rajan et al., 2000). A year after Twik related ALkaline pH activated K^+^ channels TALK-1 and TALK-2 were described and so-named because of the high pH_o_ required for their activation (Decher et al., 2001; Girard et al., 2001). On the basis of its pH_o_-dependence and on phylogenetic affinity TASK-2 is a TALK channel (Enyedi and Czirják, 2010). However, the name has stuck and despite the advice by the International Union of Pharmacology to call it K_2P_5.1 (Goldstein et al., 2005), TASK-2 continues to be the common, if informal, form of address of K_2P_5.1 (Figure 1).

## Molecular aspects of Task-2 gating

### Gating processes of K^+^ channels

K^+^ channel gating has been extensively studied by functional experiments that have received confirmation from the resolution of structures of several K^+^ channels in various states of opening (Yellen, 2002). Three different types of gating mechanisms have been proposed. A first mechanism mediates the opening and closing of K^+^ channels by changes in membrane potential and has been identified structurally with changes in the position of the inner helices lining the conduction pathway between the selectivity filter (SF) and the intracellular opening of the permeation pathway. Structures corresponding to the open and closed state have been found in KcsA and MthK K^+^ channels crystallized under appropriate conditions. The closed state corresponds to a KcsA K^+^ channel crystallized in what was deemed to be the closed state (Doyle et al., 1998). In this structure the four inner helices lie straight and bundle together toward their intracellular ends forming a narrowing lined with hydrophobic amino acids. This so-called hydrophobic seal impedes the movement of K^+^ ions (Armstrong, 2003). A structure of MthK K^+^ channel is thought to correspond to the open state and shows the inner helices bending at a hinge point, a conserved glycine located roughly half way down the helix, undoing the hydrophobic seal and creating a wide unobstructed pathway to ion passage (Jiang et al., 2002). This type of gating, sometimes identified as lower or inner gate, has been believed to be conserved in most K^+^ channels because of the widespread conservation of the glycine hinge, including channels of the K_2P_ family where it is present in helices TM2 and TM4 (Niemeyer et al., 2006).

A second type of gating occurring in various K^+^ channels is thought to take place at the SF, might involve a profound change in its structure (Yellen, 2002) and corresponds to what has been termed C-type inactivation (Hoshi et al., 1991). C-type inactivation probably is equivalent to the deformation of the SF of the KcsA channel that has been seen when these channels are crystallized in very low K^+^ concentrations (Zhou et al., 2001). Opening and closing of various K_2P_ channel has been demonstrated to entail extracellular K^+^-dependent C-type inactivation (Cohen et al., 2009) and this gating mode has been termed upper or outer gate or gating at the SF. A third mode of gating known as ball-and-chain inactivation appears absent from K_2P_ channels.

### Gating of Task-2 by extracellular pH

TASK-2 is activated by alkalinization with a p*K*_1/2_ (pH_o_ at which half of the maximal activity is attained, identical to pIC_50_) of 8.3 (Reyes et al., 1998), or 8.0 when most of the Cl^−^ in the extracellular solution is replaced by sulphate (Niemeyer et al., 2007). Decreasing pH_o_ to 6.0 abolishes TASK-2 channel activity and the pH_o_-dependence does not exhibit cooperativity. When measured in symmetrical high K^+^ solutions the single channel conductance of TASK-2 is around 60 pS at negative potentials, with reports of either no rectification or weak inward rectification as the membrane is depolarized, and gating by pH_o_ occurs by changes in its open probability (P_o_) rather than in single channel current (Reyes et al., 1998; Kang and Kim, 2004; Niemeyer et al., 2007).

A group of five charged residues residing in the large TM1-P1 extracellular loop was originally proposed to act as a sensor for pH_o_-gating in TASK-2 (Morton et al., 2005). This view was challenged (Niemeyer et al., 2006) and it was later shown that activation of TASK-2 by extracellular alkalinization was instead mediated by neutralization of R224 located near the second pore domain (Niemeyer et al., 2007). It was found that in the protonated form R224 might decrease occupancy of the SF by K^+^ leading to a blocked state. This pH_o_-dependent gating of TASK-2 at the SF was speculated to occur by the type of occupancy-related changes in the pore structure discussed above. More recently the free-energy profiles delineating ion permeation in the SF have been studied *in silico* (Zúñiga et al., 2011). The data were compatible with a situation in which protonated R224 exerting an electrostatic effect on the filter would increase the height of energy barriers between binding sites impeding ion movement without provoking pore collapse.

**Figure 2:**
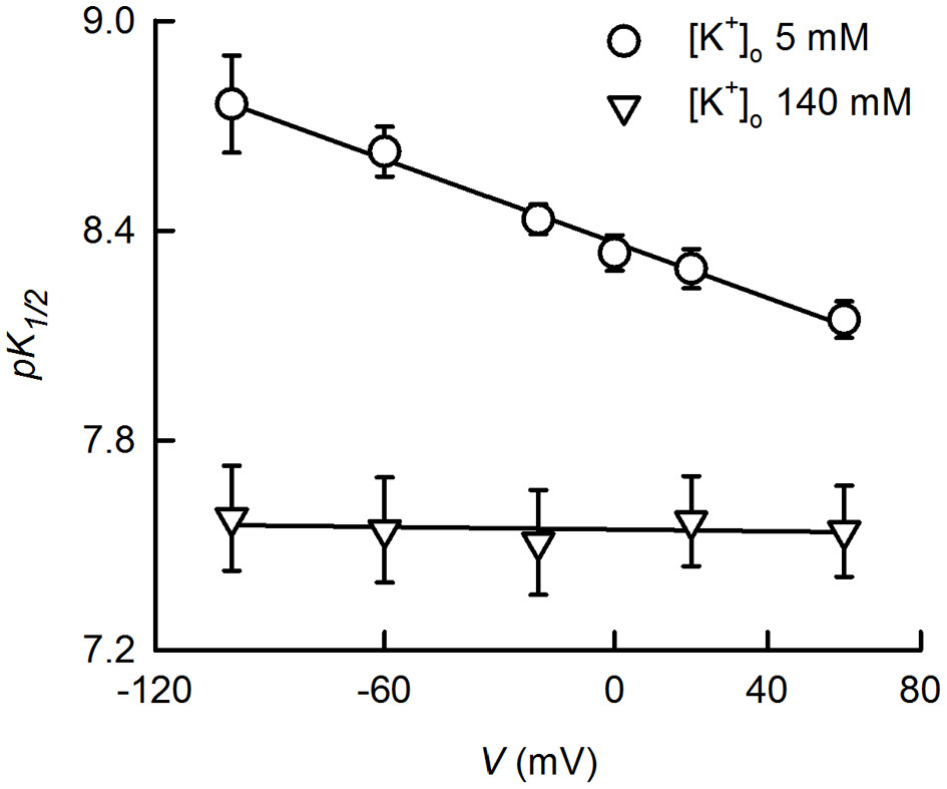
**K^+^- and voltage-dependence of TASK-gating by pH_o_**. The dependence of K^+^ currents upon extracellular pH was studied on HEK-293 cells previously transfected with TASK-2 cDNA. Measurements were done in the whole-cell recording mode of the patch-clamp technique as previously described (Niemeyer et al., 2007). The intracellular solution contained 140 mM K^+^ whilst extracellular K^+^ was either 5 or 140 mM. K^+^ 5 mM bath solution contained 135 mM sodium gluconate, 1 mM potassium gluconate, 4 mM KCl, 2 mM CaCl_2_, 1 mM MgCl_2_, 105 mM sucrose, 10 mM HEPES/Tris, pH 7.5. Intracellular, pipette solution contained 8 mM KCl, 132 mM potassium gluconate, 1 mM MgCl_2_, 10 mM EGTA, 1 mM Na_3_ATP, 0.1 mM GTP, 10 mM HEPES/Tris, pH7.4. Extracellular pH-dependence curves were generated at various voltages and fits of the Hill equation yielded the p*K*_1/2_ values reported which are averages of fitted parameters of the individual experiments. Some of the experiments for the data presented here have been published before (Niemeyer et al., 2010). Use of the Woodhull (1973) model [p*K*_1/2_(V) = p*K*_1/2_(0 mV)-zδ FV/2.303RT, where p*K*_1/2_(V) and *K*_1/2_(0 mV) are the −log of the inhibitory dissociation constants at a given voltage V and at 0 mV; δ is the fractional distance across the electric field crossed by H^+^; and z, R, T, and F have the conventional meaning], on the data at 5 mM K^+^, resulted in a δ-value of 0.22 and p*K*_1/2_(0 mV) of 8.37.

An interesting observation already made by Reyes et al. (1998), is that the p*K*_1/2_ of TASK-2 measured under quasi-physiological K^+^ concentration gradient ([K^+^]_i_/[K^+^]_o_ ~140/5 mM) is voltage-dependent. Indeed, reported p*K*_1/2_ values were 8.6, 8.3 and 7.8 at −50, 0 and 50 mV, respectively. We have more recently confirmed these results (Niemeyer et al., 2010), which are presented in more detail in Figure 2 here. It is seen that p*K*_1/2_ values vary between 8.76 at −120 mV to 8.15 at 60 mV. Voltage dependence by charged compounds acting on ion channels has often been interpreted on the basis of a model used by Woodhull to describe H^+^ blockade of Na^+^ channels (Woodhull, 1973) and has been applied to pH_o_-gating of TASK-1 channel (Lopes et al., 2001). The interpretation is that inhibition arises from the H^+^ interacting with a site located within the electric field of the membrane producing a blocked channel. Application of the Woodhull model to the TASK-2 data in Figure 2 would appear consistent with a site of action located ~20% into the membrane electric field. This does not seem to be the case for TASK-2, however, as the same experiment performed in symmetrical high K^+^ concentrations ([K^+^]_i_/[K^+^]_o_ 140/140 mM) did not show any dependence on membrane potential. How can the voltage and K^+^-dependence of pH_o_-gating of TASK-2 arise? This might occur by an electrostatic effect of K^+^ ions in the SF favoring deprotonation of charged R224 pH_o_-sensing residues (Niemeyer et al., 2010). Highest occupancy would occur under [K^+^]_i_/[K^+^]_o_ 140/140 mM conditions which would give lowest p*K*_1/2_ for pH_o_ gating without effect of membrane potential under this symmetrical high K^+^ condition. Depolarization in [K^+^]_i_/[K^+^]_o_ 140/5 mM, and therefore promoting flux of K^+^ from a side of high to one of low concentration, might increase occupancy and the electropositive influence of K^+^ on R224 sensors leading to the depolarization-dependent decrease in p*K*_1/2_. In addition, a peculiar arrangement of a so called extracellular ion pathway (EIP) unveiled by recently solved structures of K_2P_ channels TRAAK and TWIK-1 might also contribute to electrostatic interactions between K^+^ and pH_o_-sensing arginines in TASK-2.

X-ray structures for TRAAK (Brohawn et al., 2012) and TWIK-1 (Miller and Long, 2012) K_2P_ channels confirmed the homodimeric arrangement of these proteins and revealed the presence of an extracellular cap formed by the conserved TM1-P1 linkers that impedes direct access of ions into the SF to the plane of the membrane. Ion access to the pore is instead afforded by bilateral tunnel-shaped structures, the extracellular ion pathway (EIP). We have recently proposed that these entrances are central in determining the gating properties of TASK-3, a K_2P_ channel also gated by changes in extracellular pH (González et al., 2013). Molecular modeling of TASK-2 based on the TRAAK structure yields a model structure illustrated in Figure 1. Under the extracellular cap and depicted in blue is TASK-2 EIP. Interestingly, charged sensing R224 side chains seem to snorkel toward EIP. The study of TRAAK and TWIK-1 structures revealed K^+^ binding sites S1-S4 as in other non-K_2P_ K^+^ channels. An extracellular S0 or S_ext_ site, centrally located in the EIP, was also discerned both in TRAAK and TWIK-1 (Brohawn et al., 2012; Miller and Long, 2012). Additional ions are detected in the TRAAK study, associated with EIP in lateral ion access/egress positions within the EIP. K^+^ ions at these additional “binding sites” sites, presumably also present in the TASK-2 EIP, would also influence electrostatically R224 sensors contributing to the dependence of p*K*_1/2_ of pH_o_-gating upon extracellular K^+^ concentration and depolarization in low [K^+^]_o_.

### Gating of Task-2 by intracellular pH

TASK-type K_2P_ K^+^ channels are gated by extra- but not intracellular pH (Enyedi and Czirják, 2010). In contrast TALK-subfamily K_2P_ K^+^ channels TASK-2 and TALK-2 are quite sensitive to changes in pH_i_ (Niemeyer et al., 2010). Gating of TASK-2 by pH_i_ occurs in the same range of pH as for pH_o_-gating but does not require its machinery. Indeed TASK-2-R224A, which is pH_o_-insensitive, had a normal pH_i_-dependence. Early C-terminus residue K245 was identified as possible sensor for pH_i_ by a strategy involving deletions and single-point mutations. That K245 might be a genuine sensor of intracellular pH is suggested by the fact that whilst TASK-2-K245A mutant is pH_i_-insensitive, TASK-2-K245H showed an acid-shift p*K*_1/2_ of about 1 pH unit, consistent with the difference in basicity between Lys and His.

It would appear that separate gates mediate pH_o_- and pH_i_-gating of TASK-2, based on the fact that they seem to operate independently, and on the independence of pH_i_-gating on voltage and [K^+^]_o_, both characteristic of pH_o_-gating (Niemeyer et al., 2010). Largely based on the location of the pH_i_-sensor and the apparent differences between pH_i_- and pH_o_-gating, we have speculated that the gate affected by intracellular pH must correspond to the so-called lower gate (Niemeyer et al., 2010). The existence of separate upper or SF and inner or inner helices bundle crossing gating mechanisms in K_2P_ channels is, however, a matter of present controversy. Experiments involving functional analysis of mutated Drosophila KCNK0 K_2P_ channel and chimeras with the Shaker K_V_, suggest the presence of a functional lower gate in K_2P_ channels (Ben-Abu et al., 2009). Nevertheless, this lower activation gate appears mostly open as amino acids that might have been part of the hydrophobic seal of the inner helices bundle crossing are replaced by glycines, and the opening of the lower gate is coupled to the opening of the SF gate. More recently, experiments exploring the access of inhibitors to the inner vestibule of TREK-1 channel suggest that gating by intracellular pH and pressure, previously thought to occur at an inner helix bundle-crossing lower gate, occurs probably exclusively at the SF (Piechotta et al., 2011). Independent evidence also shows that although thermal- and pH_i_-sensing elements are contained within TREK-1 intracellular C-terminus, this sensing is probably transduced into gating at the SF, thus separating sensing and effector elements of the gating process (Bagriantsev et al., 2011, 2012).

### G protein modulation of Task-2 activity

Recent work has revealed that in addition to its gating by extra and intracellular pH, TASK-2 channels are inhibited by Gβγ subunits of heterotrimeric G protein (Añazco et al., 2013). G protein modulation involves physical interaction between the subunits and the channel, as suggested by coimmunoprecipitation and membrane yeast two hybrid assays identifying channel association with Gβ1 and Gβ2. Both the functional G protein effect and the subunit-channel interaction are dependent on certain key di-lysine residues in TASK-2 C-terminus. Signaling via an as yet to be identified G protein-coupled receptor could be a way for hormonal regulation of TASK-2 in the kidney although actions of Gα and Gβγ can occur both by receptor-dependent and -independent processes (Blumer et al., 2007). There is no clarity as to what gate, internal or external, is affected by Gβγ action, but the effect seems independent of pH_o_- and pH_i_-gating. The only previous evidence for direct modulation and physical interaction between a K_2P_ channel and Gβγ subunits of G protein is provided by regulation of TREK-1 that participate in glutamate release in astrocytes (Woo et al., 2012). The effect of Gβγ is to change dramatically TREK-1 selectivity, which becomes highly glutamate permeable.

## Physiological functions of Task-2

TASK-2 expression has been reported in a variety of cells and tissues ranging from kidney to immune cells and including specific neurons, its proposed functions spanning from an involvement in the regulation of cell volume to the control of excitability. Some of the most prominent functions proposed for TASK-2 are examined below.

### Regulatory volume decrease

Most cells are capable of adjusting their volume on the face of acute changes in intra- or extracellular osmotic pressure followed by rapid fluxes of water across their plasma membrane (Hoffmann and Pedersen, 2011). Osmotically swollen cells release KCl and organic osmolytes such as taurine, with a concomitant osmotically-forced loss of cell water leading to a reduction in volume and recovery toward pre-swelling values. This process has been termed regulatory volume decrease (RVD) and it is often achieved by parallel activation of osmosensitive K^+^ and Cl^−^ channels. TASK-2 channels have been shown to be involved in RVD of Ehrlich cells (Niemeyer et al., 2001), a classical model used in cell volume regulation field (Hoffmann and Pedersen, 2011). In these cells, a K^+^ channel insensitive to Ca^2+^ but activated by osmotic cell swelling and termed I_K, vol_ is a functional correlate of TASK-2 (Niemeyer et al., 2000). TASK-2 expressed in HEK-293 cells responds to changes in cell volume and enhances the RVD response (Niemeyer et al., 2001). The involvement of native TASK-2 channels in RVD has also been shown in mouse renal proximal tubule cells using TASK-2 KO mice (Barrière et al., 2003) and TASK-2 channels have also been implicated in apoptotic volume decrease (AVD) that precedes cell death (L'Hoste et al., 2007b). Other cells where TASK-2 has been proposed to play a role in RVD are spermatozoa (Barfield et al., 2005), retinal Müller glial cells (Skatchkov et al., 2006) and T-lymphocytes (Bobak et al., 2011).

The mechanism underlying acute RVD lacks a satisfactory, unifying formulation (Hoffmann and Pedersen, 2011). In particular, although hypotheses for activation of TASK-2 during RVD have been advanced, these remain to be substantiated and completed particularly regarding the identity of the sensor of cell volume change and its coupling to channel activity. In kidney cells it has been proposed that cell swelling-dependent TASK-2 activation is consequent to extracellular alkalinization evoked by enhanced Cl^−^ influx through the Cl^−^/HCO^−^_3_ exchanger. A demonstration of the increased Cl^−^ influx, however, is lacking (L'Hoste et al., 2007a). RVD in Ehrlich cells is decreased by inhibition of tyrosine phosphorylation, an effect that might be mediated by direct tyrosine kinase-dependent phosphorylation of TASK-2 (Kirkegaard et al., 2010). The TASK-2 tyrosine residue(s) involved have not been identified and a demonstration that the activity of the channel can be modulated by this phosphorylation has not been tested by electrophysiological recordings of TASK-2 activity. In Ehrlich cells it has been shown that leukotriene D4 (LTD_4_) is released upon hypotonic stress (Lambert, 1987) and LTD4 activates a K^+^ conductance similar to I_K, vol_ (Hougaard et al., 2000). There is, however, no evidence for direct LTD_4_ regulation of TASK-2. Also working on Ehrlich cells we have shown that an unidentified G protein is involved in the regulation of I_K, vol_. After activation by hypo-osmotic cell volume increase the decrease in I_K, vol_ in isotonicity is accelerated by GTP-γ-S but impeded by GDP-β-S, consistent with I_K, vol_ being inhibited by an active G protein that contributes to channel closure after cell volume recovery (Niemeyer et al., 2002). Recently it has been shown that neutralization of lysine pairs in the C-terminus region of TASK-2 that are essential for Gβγ inhibition, impairs the ability of the channel to respond to a cell volume change (Añazco et al., 2013). The result could be interpreted to suggest that G-protein modulation of TASK-2 is part of the mechanism by which the channel responds to cell volume changes. There have been suggestions that a G protein could be targeted by a cell volume signal, perhaps by coupling to a cell volume sensor (Davis et al., 1992; Margalit et al., 1993; Ishii et al., 1996), but the direct evidence of this contention is lacking.

### Task-2 and renal bicarbonate reabsorption

Buffering intra- and extracellular pH is a central homeostatic function. Bicarbonate is the main buffer controlling blood pH and its levels are under careful control by the renal and respiratory systems. As briefly summarized in this and the following section, experiments using TASK-2 null mice have shown a central role for TASK-2 in proximal tubule bicarbonate reclaim process (Warth et al., 2004) as well as in the signaling in central CO_2_ and O_2_ sensitive neurons which contribute to the control of breathing (Gestreau et al., 2010). These results have been reviewed recently (Lesage and Barhanin, 2011) and will only be discussed briefly here.

Most of the bicarbonate of the glomerular filtrate is salvaged in the proximal tubule by mechanisms that by and large have been elucidated (Skelton et al., 2010). Under the action of carbonic anhydrase (CA) IV, HCO^−^_3_ together with H^+^ secreted into the lumen produce CO_2_ and H_2_O. CO_2_ finds its way into the cell where CA II catalyzes the hydration of CO_2_ to produce HCO^−^_3_. Intracellular HCO^−^_3_ is then transported into the peritubular plasma by the electrogenic Na^+^/HCO^−^_3_ cotransporter (NBCe1-A) with a 3-to-1 HCO^−^_3_-to-Na^+^ stoichiometry. Proximal tubule cell have to respond to the changing demands in acid base regulation of the organism to regulate HCO^−^_3_ reabsorption accordingly (Brown and Wagner, 2012). Dysregulation will lead to disease as in pRTA, proximal renal tubular acidosis (Alper, 2010). TASK-2 is expressed in proximal tubule epithelium and TASK-2 KO mice present metabolic acidosis and hypotension secondary to renal loss of HCO^−^_3_ (Warth et al., 2004). Reabsorption of HCO^−^_3_ requires the activity of a basolateral K^+^ channel to serve the purposes of recycling K^+^ taken up in the pumping cycle of the Na^+^-K^+^ pump and to maintain a hyperpolarized membrane potential compatible with NBCe1-A-mediated outward flux. It is proposed that TASK-2 keeps pace with NBCe1-A activity through extracellular basolateral alkalinization brought about by the very HCO^−^_3_ efflux. Evidence for this model of HCO^−^_3_ transport regulation is lacking in intact proximal tubules, and it has been proposed that regulation is exerted instead by basolateral CO_2_ and HCO^−^_3_ sensing (Zhou et al., 2005). Whilst CO_2_ and HCO^−^_3_ might be sensed in the kidney to gauge the general organism acid-base balance, the idea of extracellular pH in a microdomain serving to couple NBCe1-A and TASK-2 to ensure homeostasis at the epithelial cell level is very attractive. Direct measurement of pH in the putative microdomain, perhaps by targeted sensors (Urra et al., 2008), will be required to test the hypothesis.

### Task-2 and central chemoreception

Changes in CO_2_ and/or pH in the brain stem serve as stimuli to control breathing. Neurons that regulate breathing rate and intensity and active expiration, and are therefore critical for the regulation of CO_2_ and pH, reside in the retrotrapezoid nucleus (RTN) and have been identified by functional and genetic modification experiments (Dubreuil et al., 2009; Guyenet and Mulkey, 2010). Sensing of CO_2_ by RTN nucleus neurons has been proposed to be the consequence of regulation of leak K^+^ channels by extra- or intracellular pH changes acting as CO_2_ proxies and affecting excitability, but the channel involved had not been identified (Mulkey et al., 2007). TASK-2 is found in the mouse brain only in a small number of cells in brainstem nuclei, including the RTN. Transgenic mice expressing a mutated Phox2b gene, coding for the Paired-like homeobox 2b transcription factor, associated to human congenital central hypoventilation syndrome suffer from a lack of CO_2_ chemosensitivity and the disappearance of RTN neurons that express TASK-2 (Dubreuil et al., 2008). TASK-2 KO mice show disturbed respiratory responses to hypoxia and hypercapnia and it has been suggested that TASK-2 channels might be an essential component of the chemoreceptive apparatus of the RTN neurons (Gestreau et al., 2010). Reactive oxygen species (ROS) have been proposed as possible agents in hypoxia modulation of TASK-2 in RTN neurons. TASK-2 activation has been shown to take place in heterologous expression systems by xanthine/xanthine oxidase treatment (Duprat et al., 2005; Papreck et al., 2012). On the other hand, TASK-2 sensitivity to extra- and/or intracellular pH might play roles in a putative function of the channel as a pH/CO_2_ sensor in RTN neurons, particularly since the last property would allow TASK-2 to behave as a CO_2_ sensor (Niemeyer et al., 2010). It must be pointed out that the role of TASK-2 in central chemosensing has been put in doubt (Chernov et al., 2010; Guyenet et al., 2010). It is argued that TASK-2 ablation, in contrast to what is seen after deletion of RTN neurons, does not produce the expected alteration in pH sensitivity of the neonate breathing network *in vitro*. Also because the renal effect of TASK-2 ablation, leading to impaired bicarbonate retention, the mice are in metabolic acidosis which itself might alter their central response to CO_2_. It is suggested that the TASK-2 channel, though involved, is not the sole chemosensory ion channel since KO animals had a definite ventilatory response to hypercapnia. It seems therefore that the proverbial further work is needed to establish its status in chemoreception, perhaps by direct electrophysiological recording of RTN neurons.

### Expression and possible functions of Task-2 in chondrocytes

Hyaline cartilage is found on articular surfaces and in the trachea. The role of hyaline chondrocytes is to support the cartilage structure through the generation of its extracellular matrix that must withstand mechanical loads whilst maintaining elasticity. Ion channels and in particular K^+^ channels are viewed as important players in chondrocyte function. A 2010 review dealing with the so-called “chondrocyte channelome” cites the presence of various kinds of K^+^ channels, proposed to be involved in the regulation of chondrocyte membrane potential, regulating metabolic activity, coupling membrane stretch to RVD and intracellular Ca^2+^ regulation (Barrett-Jolley et al., 2010). K_2P_ channels were conspicuously absent from the list. Expression of TASK-2 in human articular chondrocytes, however, was reported shortly afterwards (Clark et al., 2011). Acutely cultured chondrocytes express TASK-2 as assessed by RT-PCR and immunolocalization, and present TASK-2-like channel activity in electrophysiological experiments. TASK-2 is demonstrated to play a central role in stabilizing chondrocyte membrane potential. In addition, TASK-2 expression has also been reported in a study of osteoarthritis, where it is shown to decrease markedly with the progression of the disease (Karlsson et al., 2010; Mobasheri et al., 2012).

**Figure 3:**
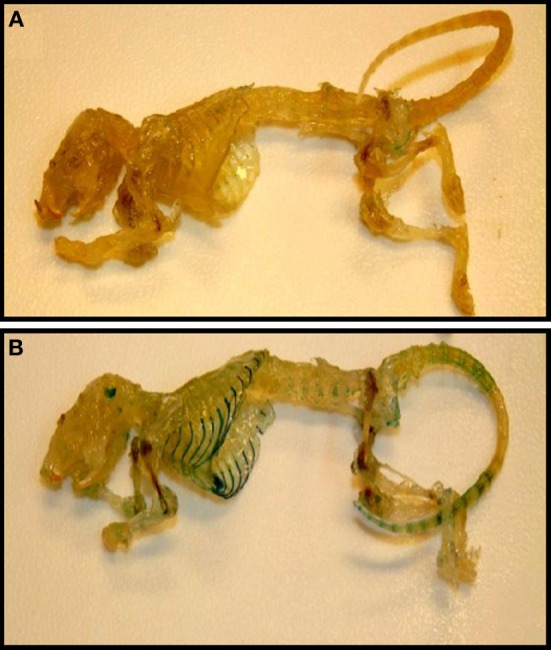
**Cartilage as a site of TASK-2 expression revealed by β-galactosidase activity in TASK-2 KO mice**. Skeletons from WT **(A)** and TASK-2 KO **(B)** mice were prepared by clearing and decalcification followed by staining for X-gal (blue). The KO mouse was generated by a gene-trap approach where the trapping vector encodes a β-galactosidase/neomycin resistance fusion protein (Araki et al., 2009). The enzyme is expressed under the control of the TASK-2 promoter and therefore the β-galactosidase activity reflects sites of endogenous TASK-2 gene expression.

**Figure 4:**
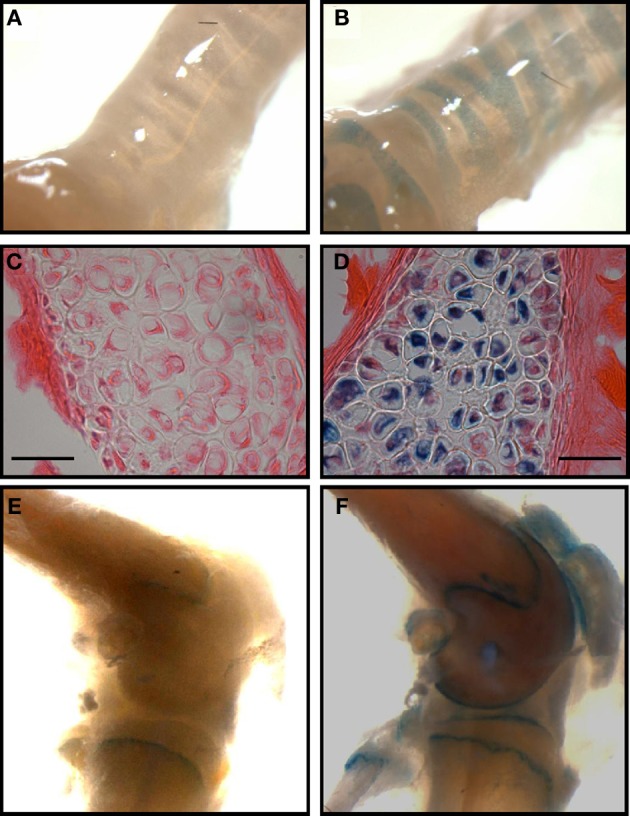
**TASK-2 expression in hyaline cartilage from trachea and articular surface of the knee. (A,B)** are images of whole trachea stained with X-gal in wild type and heterozygous TASK-2^(+/−)^ mice, respectively. The signal is present in the cartilage rings. In **(D)** the image demonstrates staining in chondrocytes [**(C)**: wild-type control]. **(E,F)**: images obtained from control and heterozygous TASK-2^(+/−)^ mouse knees. The macroscopic staining in the area of the epiphyseal growth plate observed in WT knee corresponds to cells that are expressing endogenous mammalian ß-gal activity, most likely osteoclasts (Kopp et al., 2007). Bars in **(C,D)** correspond to 25 μm.

The presence of TASK-2 being clearly demonstrated in cartilage (Karlsson et al., 2010; Clark et al., 2011; Mobasheri et al., 2012), we take advantage of the TASK-2 KO animal to illustrate its presence in mouse articular cartilage. The mouse used was generated using a trapping vector encoding a β-galactosidase that is now under the control of the TASK-2 promoter, its activity reflecting the endogenous TASK-2 gene expression (Taniwaki et al., 2005). Skeleton preparations show the presence of TASK-2 at cartilage locations (Figure 3). Particularly strong stain is observed in the rib cage compatible with TASK-2 expression in costal hyaline cartilage. Figures 4A,B show whole isolated trachea from WT (A) and heterozygous TASK-2 KO (B) mice after X-Gal staining, showing a strong signal in cartilage rings. Histological examination shows the expression to occur in chondrocytes (Figures 4C,D). Other hyaline cartilage also presented evidence of TASK-2 expression. An example is given in Figures 4E,F that shows knee articular cartilage. TASK-2 has been proposed to contribute to setting chondrocyte membrane potential and it could be a major player in linking extracellular pH and cell activity. A comparative study of WT and TASK-2 KO mice could help in understanding the role of the channel in cartilage physiology and pathophysiology.

### A role for Task-2 in lymphocyte biology?

The function of K^+^ channels in lymphocyte biology is well-established (Cahalan and Chandy, 2009) with voltage-gated Kv1.3 (KCNA3) and the Ca^2+^-dependent IK_1_ (KCNN4) channel known to be central in the Ca^2+^ signaling of T and B lymphocyte activation. K_2P_ K^+^ channels made their debut in lymphocyte publications with the report of two background-type K^+^ channels in WEHI-231 cells, a B lymphocyte-type cell from murine lymphoma (Nam et al., 2004) and have later been suggested to play roles in various immunological processes (Flores et al., 2011). An upregulation of TASK-2 in CD4 and CD8 T cells has been shown in relapsing/remitting Multiple Sclerosis (MS) patients (Bittner et al., 2010a). TASK-2 was also identified in WEHI-231 immature B cells where it is activated by stimulation of B cells receptors (BCR ligation) and participates in BCR-ligation-dependent apoptosis (Nam et al., 2011). The process of apoptosis is central to the prevention of autoimmune reaction and in the maintenance of lymphocyte populations and its dysregulation can lead to immunodeficiency, autoimmune disease or malignant growth (Maniati et al., 2008). It will be interesting to confirm the presence of TASK-2 in human B cells. In addition, TASK-2 KO mice could be used convincingly to confirm the expression of TASK-2 in lymphocytes and serve as good models in which to explore possible immunological phenotypes associated to channel ablation.

### Other possible functions of Task-2

Sightings of TASK-2 in other cells and tissues than those reviewed above have been reported. The possible function of TASK-2 in these locations awaits elucidation by further studies.

Using TASK-2-promoter driven β-galactosidase activity of gene-trap KO mice TASK-2 has been shown to be expressed in both endocrine and exocrine pancreas (Duprat et al., 2005). It is speculated that together with related K_2P_ family members TALK-1 and TALK-2, TASK-2 might play a non-specified role in exocrine pancreas function.

On the basis of RT-PCR, immunocytochemistry and electrophysiological assays TASK-2 has been proposed to be present in rat dorsal root ganglion (DRG) neurons (Kim et al., 2012). TASK-2 is reported to be developmentally-regulated in DRG and is suggested to play a predominant role in cell excitability in neonatal rather than adult DRG neurons.

RT-PCR and immunocytochemistry also suggest that TASK-2 might be present in intestinal smooth muscle cells where it could contribute significantly to setting the resting membrane potential and to the regulation of excitability (Cho et al., 2005).

The expression of TASK-2 has been reported in certain cell lines of human breast tumor origin whose proliferation is estrogen-dependent through an effect on the estrogen α receptor (ERα). A recent paper reports the presence of TASK-2 in two of these ERα^+^ cell lines and the 17β-estradiol (E2) dependent increase in the channel mRNA and protein (Alvarez-Baron et al., 2011). TASK-2 gene enhancer possesses estrogen-responsive elements and is capable of binding the ERα after E2-treatment of the cells. TASK-2 is proposed to play a role in regulating the proliferation of breast cancer cells and could therefore be considered a candidate target for breast cancer therapeutics.

### Task-2 in pathological conditions

The role TASK-2 might play in pathological states has been recently considered in a review (Bittner et al., 2010b). Besides the possible involvement of TASK-2 in MS mentioned above, upregulation of TASK-2 could play a role in protection against cellular stress and, in experimentally induced temporal lobe epilepsy, in dampening of epileptic activity (Kim et al., 2009).

### Concluding remarks

Since the discovery of TASK-2 by Reyes et al. (1998), we have steadily come to the realization that it is endowed with complex regulation that might tune its activity to physiological functions. TASK-2 is only partially, around 25%, open at physiological pH. It is gated by both intra- and extracellular pH having plenty of scope for activation by alkalinization. The property of intracellular pH-gating is shared by TASK-2 congeners of the TALK but not the TASK subfamily of pH-sensitive K_2P_ channels. In addition TASK-2 physically interacts with Gβ subunits and appears regulated by a Gβγ complex. Extra- and intracellular pH have been proposed to affect separate gating machineries within the channel, but recent work challenging the existence of these two gates in K_2P_ channels militates against this concept. Sensors for intra- and extracellular pH have been identified and correspond in the mouse to lysine 245 and arginine 224, respectively. Using a knockout mouse expressing β-galactosidase TASK-2 expression is reported in kidney, exocrine and endocrine pancreas, a reduced number of specific neurons and hyaline cartilage. TASK-2 has been shown to participate in the so-called regulatory volume decrease, in which osmotically swollen cells recover their volume by KCl loss through selective channels, or in a similar phenomenon essential to apoptosis. Experiments using TASK-2 knockout mouse suggest an involvement of the channel in renal bicarbonate reabsorption as well as signaling processes in central CO_2_ and O_2_ sensitive neurons which contribute to the control of breathing. Less well-documented functions of TASK-2 are proposed for the regulation of chondrocyte membrane potential and metabolic activity, the maintenance of lymphocyte populations by apoptosis, the control of cell excitability in neonatal dorsal root ganglion neurons, and the regulation of excitability in intestinal smooth muscle cells. Interestingly TASK-2 might play a central role in the regulation of proliferation in breast estrogen-dependent cancer cells, making it a putative target in cancer therapeutics. Despite advances in our understanding of molecular aspects of TASK-2 function there is still much to be learned about how cell signals tune the activity of TASK-2 to its specific functions. The availability of newly K_2P_ structural characteristics will no doubt help in this respect. Future work in native tissues and cells exploiting available and future genetically-modified animals will certainly reveal in detail the functions of this channel that we are only starting to understand.

### Conflict of interest statement

The authors declare that the research was conducted in the absence of any commercial or financial relationships that could be construed as a potential conflict of interest.
